# Operant and classical learning principles underlying mind–body interaction in pain modulation: a pilot fMRI study

**DOI:** 10.1038/s41598-021-81134-6

**Published:** 2021-01-18

**Authors:** In-Seon Lee, Won-Mo Jung, Ye-Seul Lee, Christian Wallraven, Younbyoung Chae

**Affiliations:** 1 Acupuncture and Meridian Science Research Center, College of Korean Medicine, Kyung Hee University, 1 Hoegi-dong, Dongdaemun-gu, Seoul, 02447 Republic of Korea; 2 Department of Anatomy and Acupoint, College of Korean Medicine, Gachon University, Seongnam, Republic of Korea; 3 Department of Artificial Intelligence, Department of Brain Cognitive Engineering, Korea University, Seoul, Republic of Korea

**Keywords:** Neuroscience, Psychology

## Abstract

The operant conditioning has been less studied than the classical conditioning as a mechanism of placebo-like effect, and two distinct learning mechanisms have never been compared to each other in terms of their neural activities. Twenty-one participants completed cue-learning based pain rating tasks while their brain responses were measured using functional magnetic resonance imaging. After choosing (instrumental) or viewing (classical) one of three predictive cues (low- and high-pain cues with different level of certainty), they received painful stimuli according to the selected cues. Participants completed the same task during the test session, except that they received only a high pain stimulus regardless of the selected cues to identify the effects of two learning paradigms. While receiving a high pain stimulation, low-pain cue significantly reduced pain ratings compared to high-pain cue, and the overall ratings were significantly lower under operant than under classical conditioning. Operant behavior activated the temporoparietal junction significantly more than the passive behavior did, and neural activity in the primary somatosensory cortex was significantly reduced during pain in instrumental as compared with classical conditioning trials. The results suggest that pain modulation can be induced by classical and operant conditioning, and mechanisms of attention and context change are involved in instrumental learning.

## Introduction

The placebo effect, which occurs when an inactive treatment leads to positive outcomes, is a genuine example of mind–body interaction, as it demonstrates conscious or unconscious modulation of our brain, behavior, and physiological responses. Among various mechanisms underlying the placebo effect, including expectation, conditioning with our without conscious learning, and reward learning, the most extensively studied mechanisms are expectation (e.g., expectation of clinical improvement) and conditioning (e.g., conditioned therapeutic effect induced by placebo administration)^1–3^. The role of expectation in the placebo effect is mainly evident in verbally induced expectancy manipulation paradigms, such as suggestion of positive treatment effects^4–6^, and open-hidden administration of drugs^7–10^. While verbally manipulated expectations modulate human behaviors explicitly and consciously, classical Pavlovian conditioning changes a neutral stimulus into a conditioned stimulus, which subsequently elicits involuntary (conditioned) responses after repeated pairings with the unconditioned stimulus. Classical conditioning does not solely produce unconscious physiological changes (e.g., drug-like responses by administration of inert vehicle or by conditioned cue stimulus after repeated drug administration^1,11,12^), on the other hand, it also modulates our expectations for the environment. Previous studies have suggested that the conditioning procedure generates or alters expectation, and expectation changes the effect of conditioning on the placebo effect^3,13,14^. In summary, evidence suggests that learning, either conscious or unconscious, is a crucial mechanism underlying the placebo effect induced by a classical conditioning paradigm^15,16^.

Classical conditioning requires active processing of the predictive value of a conditioned stimulus, which elicits expectations and results in the acquisition of a conditioned response^17^. Operant conditioning (also called instrumental conditioning) is another type of learning procedure that involves a reinforcer or punisher, which increases or reduces, respectively, the frequency of a voluntary behavior. The classical conditioning paradigm has been extensively studied as a mechanism and means of generating the placebo effect since early stages of placebo research^18–21^, whereas studies of other types of learning were initiated later. Fordyce suggested that pain behaviors might occur as a reinforcement (e.g., avoiding aversive events), eventually leading to chronic pain^22^. In a series of studies by Hölzl and Becker, implicit learning of pain relief and pain increase was combined with operant conditioning (temperature-lowering behavior was rewarded, and temperature-increasing behavior was punished) in healthy^23,24^ and chronic pain patients^25^. The results showed that intrinsic operant reinforcers were effective in changing sensitization and habituation behaviors. Recently, placebo analgesia was successfully induced by operant conditioning paradigm applying the contingency between pain responses and reward/punishers^26^. As previous studies have demonstrated, pain relief is a reward and serves as a reinforcer^27–29^. Operant conditioning using extrinsic or intrinsic reinforcers/punishers leads to changes in pain perception through reward learning.

Although learning processes are related to the classical and operant conditioning-induced placebo effects, overlapping and distinct mechanisms underlying expectations and various types of learning are still under debate. Only few studies have investigated the neural substrates of operant conditioning of pain modulation^30–32^, and it still remains unrevealed how our brain is working during different types of learning tasks and placebo analgesic phase. Functional neuroimaging techniques, such as functional magnetic resonance imaging (fMRI) and positron emission tomography (PET), have revealed brain regions involved in the effect of placebo on pain perception in humans, such as anterior cingulate cortex, prefrontal cortex, and periaqueductal gray^33,34^. However, to the best of our knowledge, no studies have yet been conducted investigating disparate neural activities mediating the pain modulation effect via operant and classical learning processes.

In the current study, we aimed to test the validity of pain relief as a reinforcer of instrumental behavior in a pain modulation paradigm, and identify brain regions which are likely to have different roles in operant and classical learning processes. We hypothesized that brain activities through operant conditioning might be different from brain activities through classical conditioning, as the former requires intentional exploration of the environment and instrumental behavior. However, we did not limit our analysis to a specific region due to the lack of evidence. To test our hypothesis, we implied fMRI during operant and classical learning tasks and asked participants to choose actively (operant conditioning) or to see passively (classical conditioning) predictive visual cues, which suggest the amount of pain relief, and rate the intensity of painful mechanical stimuli. We measured their learning behavior using a number of cue choices in the operant conditioning session, and the placebo-like effect was defined by the influence of visual cues (one implied that they would receive high pain stimulus while two other cues implied that they would receive low pain stimulus) on subjective pain ratings to the same noxious stimuli in both operant and classical conditioning session. Functional activities during the cue choice/viewing tasks and pain perception were compared between the operant and classical test sessions.

## Results

### Operant learning of pain relief cues

**Figure 1:**
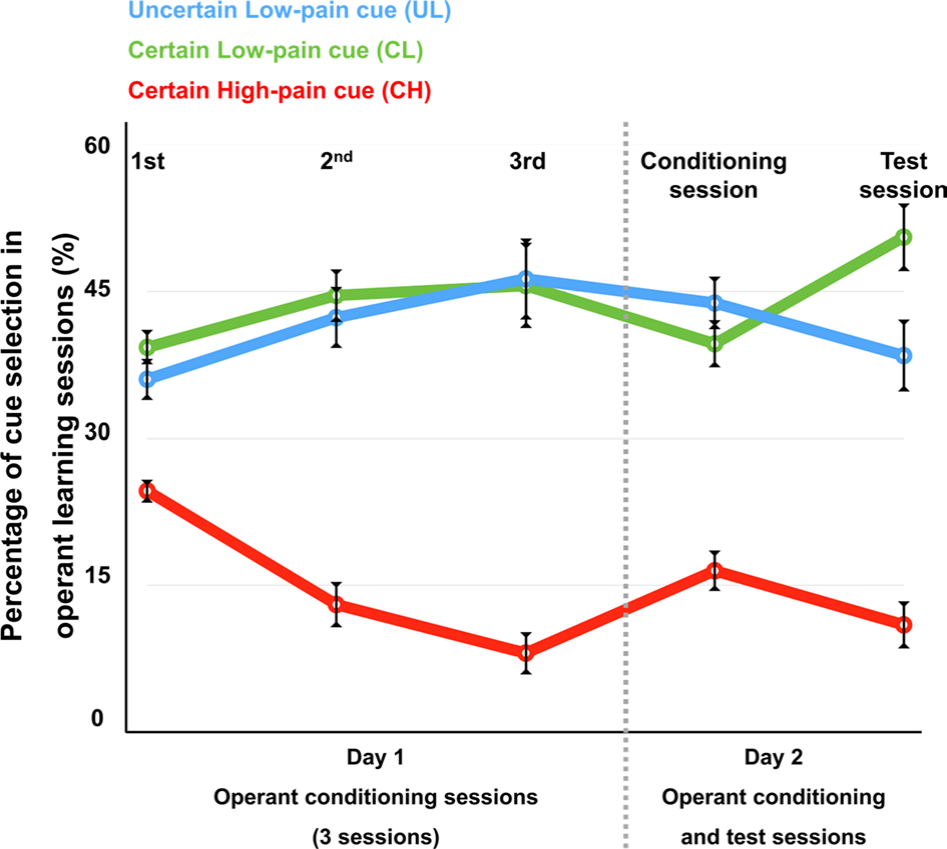
Operant learning of visual cues. Error bars represent between-subject standard errors. Participants preferred cues predicting low pain to those predicting high pain regardless of the certainty of the cues, suggesting that the participants learned the association between visual cues and pain levels through operant conditioning (UL vs. CL: *t* = −1.36, *p* = 0.54; UL vs. CH: *t* = −7.20, *p* = 3.4e−09; CL vs. CH: *t* = −5.84, *p* = 6.7e−07).

In this study, we measured participants’ learning behavior using the number of trials in which they selected each visual cue. To evaluate each participant’s choices of visual cues—the uncertain low pain (UL) cue, the certain low pain (CL) cue, and the certain high pain (CH) cue- during the cue-selection task in the operant conditioning session, we conducted a repeated-measure ANOVA test, which revealed the significant effect of visual cues on the cue choice task (*p* = 1.888e−13; Cohen’s f = 1.21, 95% confidence interval [0.78, 1.6]). Post hoc testing with the Bonferroni correction showed that both low-pain cues were significantly more frequently selected than the high-pain cue (UL vs. CL: *t* = −1.36, *p* = 0.54; UL vs. CH: *t* = −7.20, *p* = 3.4e−09; CL vs. CH: *t* = −5.84, *p* = 6.7e−07; Fig. 1).

### Intensity ratings of the second pain

Participants were asked to rate the relative magnitude of pain induced by the second painful stimulus (various pinprick needles adjusted according to the selected cues) relative to the first painful stimulus (512 mN pinprick needle) which serves as a reference pain intensity. We tested this method in our previous study, and it allowed us to minimize sensitized or habituated responses to repeated painful stimuli^35^.

For overall pain ratings, two-way ANOVA showed significant main effects of type of learning (*p* = 0.04; Cohen’s f = 0.17, 95% confidence interval [0.03, 0.30]) and visual cues (*p* = 2e−16; Cohen’s f = 0.63, 95% confidence interval [0.48, 0.77]). The post hoc t-tests with Bonferroni correction showed that the pain ratings were significantly lower in operant than in classical learning trials (operant vs. classical learning: *t* = −2.011, *p* = 0.045). The pain intensity ratings following the UL and CL cues were significantly lower than those following the CH cue, which we defined as placebo-like effect driven by learning the associations between cues and pain intensities (UL vs. CH: *t* = 7.573, *p* = 4.7e−09; CL vs. CH: *t* = 8.588, *p* = 3.8e−11). There was no significant difference in the intensity of pain between the UL and CL trials (*t* = −1.043; *p* = 0.55).

#### Conditioning session

**Figure 2:**
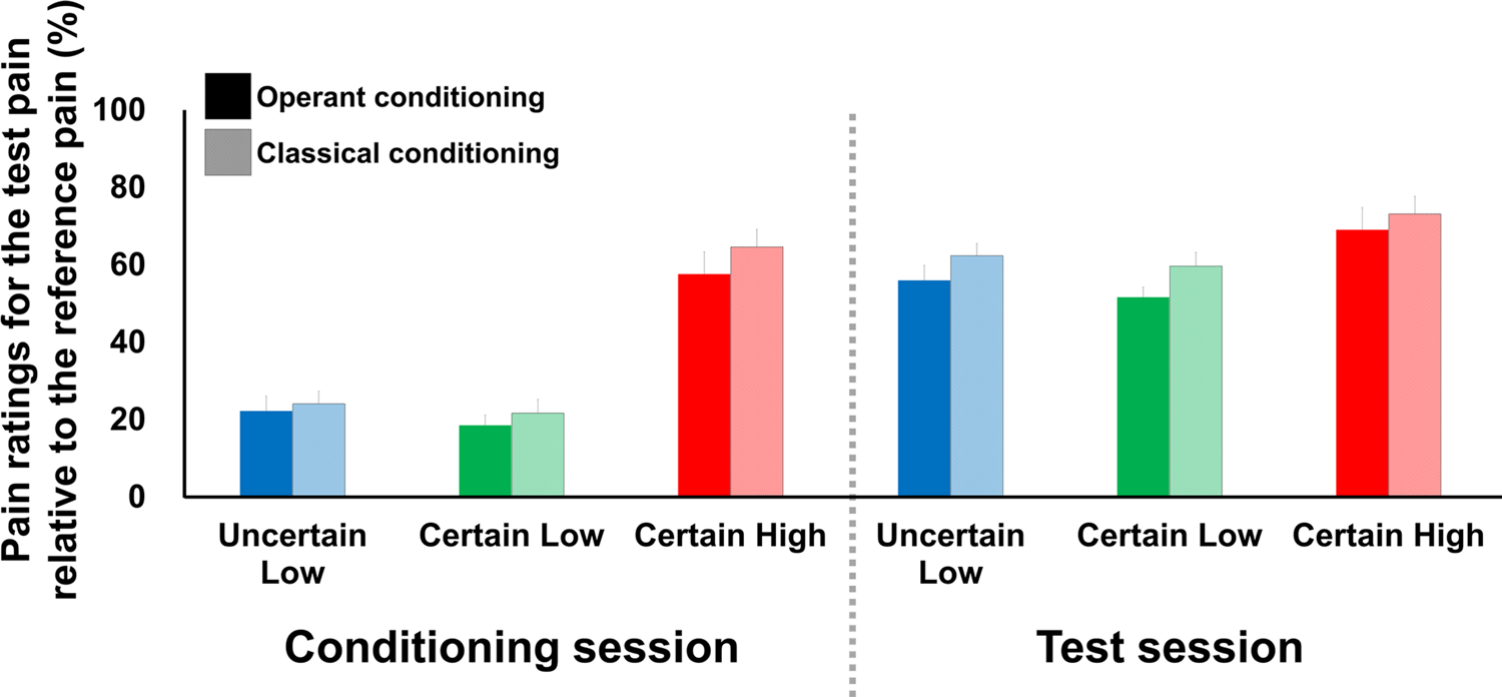
Pain ratings during the conditioning and test sessions on day 2. Bars represent the average relative pain ratings in response to the second pain stimulus relative to the reference pain, and error bars represent between-subject standard errors. Significant main effects of type of learning (*p* = 0.04; Cohen’s f = 0.17, 95% confidence interval [0.03, 0.30]) and visual cues (*p* = 2e−16; Cohen’s f = 0.63, 95% confidence interval [0.48, 0.77]) were determined by two-way ANOVA. The post hoc *t*-tests with Bonferroni correction showed that the pain ratings were significantly lower in operant than in classical learning trials (operant vs. classical learning: *t* = −2.011, *p* = 0.045). Additionally, the intensity ratings of pain experienced with the 256 mN pinprick needle following the UL and CL cues were significantly lower than those for the same painful stimulus (the 256 mN needle) following the CH cue (UL vs. CH: *t* = 7.573, *p* = 4.7e−09; CL vs. CH: *t* = 8.588, *p* = 3.8e−11). There was no significant difference in the intensity of pain between the UL and CL trials (*t* = −1.043; *p* = 0.55).

Pain intensity ratings for the second pain were 22.3 ± 3.8 (mean ± standard error; active UL), 18.5 ± 2.6 (active CL), 57.6 ± 5.7 (active CH), 24.1 ± 3.2 (passive UL), 21.7 ± 3.6 (passive CL), 64.6 ± 4.6 (passive CH) during the conditioning session. Two-way ANOVA revealed no significant, but marginal main effect of conditioning type (operant vs. classical conditioning, *p* = 0.059; Cohen’s f = 0.21, 95% confidence interval [0.00, 0.14]), but found a significant main effect of cues on the pain ratings (*p* = 2e−16; Cohen’s f = 1.85, 95% confidence interval [1.52, 2.16]) during the conditioning session on day 2. A post hoc test showed that the subjective intensities of the painful stimulus following the UL and CL cues were significantly lower than the ratings following the CH cue (UL vs. CH: *t* = −15.46, *p* = 2.3e−16; CL vs. CH: *t* = −16.70, *p* = 2e−16). The intensities of subjective pain did not differ significantly between the UL and CL trials (UL vs. CL: *t* = 1.26, *p* = 0.42; Fig. 2).

#### Test session

Pain intensity ratings for the second pain were 56.0 ± 5.2 (active UL), 51.7 ± 4.9 (active CL), 69.1 ± 4.0 (active CH), 62.4 ± 4.7 (passive UL), 89.7 ± 5.7 (passive CL), 73.2 ± 4.3 (passive CH) during the test session. With respect to the intensity of pain reported during the test session on day 2, two-way ANOVA revealed significant main effects of type of learning (*p* = 0.008; Cohen’s f = 0.32, 95% confidence interval [0.12, 0.53]) and visual cue (*p* = 1.26e−05; Cohen’s f = 0.49, 95% confidence interval [0.26, 0.69]). A post hoc test showed that the pain ratings were significantly lower in operant learning trials than in classical, thus the stimulus was perceived as less painful with operant learning than with classical conditioning (active vs. passive: *t* = −2.67, *p* = 0.009). The intensities of pain following the UL and CL cues were significantly lower than those following the CH cue (UL vs. CH: *t* = −3.50, *p* = 0.04; CL vs. CH: *t* = −4.61, *p* = 0.006), as determined by the post hoc with Bonferroni correction test. The subjective pain intensities did not differ significantly between the UL and CL trials (*t* = 1.18; *p* = 0.46; Fig. 2).

We compared the conditioning induced placebo analgesic effects by two types of learning using paired t-test for a [pain ratings in high-cue trials—those in low-cue trials] contrast of active vs passive condition, however, the pain modulation effect did not differ significantly between the operant and classical learning conditions (*t* = −0.047, *p* = 0.96).

### Brain activity during cue-selection tasks (test session)

**Figure 3:**
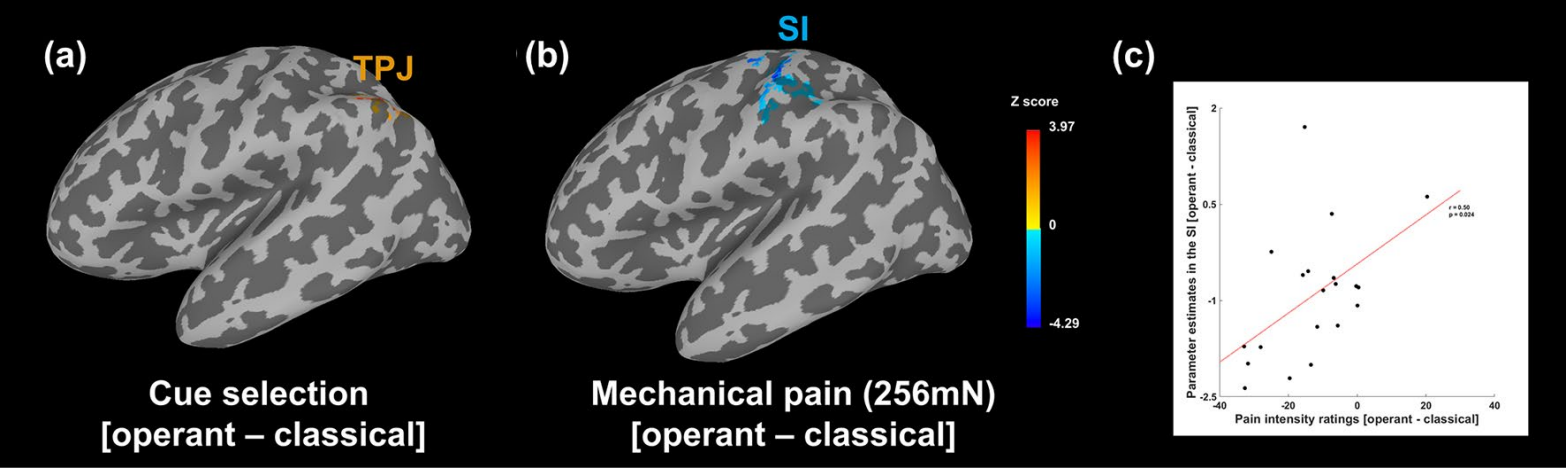
Brain responses during cue selection (**a**) and painful stimulation (**b**) (operant vs. classical learning). (**a**) Voxels in the left inferior parietal gyrus and superior temporal sulcus showed significantly greater activity during the voluntary choice of cues based on the anticipation of pain relief formed through instrumental learning compared to a simple motor task in classical conditioning (clicking the cue selected by the computer; peak coordinate: X = −45.5, Y = −60, Z = 56.5, 38 voxels, cluster-level FWE corrected *p* < 0.05). (**b**) Perception of pain following the instrumental learning cues, compared with identical painful stimulation following the classical conditioning cues, was associated with decreased activity in the left primary somatosensory cortex, primary motor cortex, and parietal lobe (peak coordinate: X = −52.5, Y = −18, Z = 60, 94 voxels, cluster-level FWE corrected *p* < 0.05). (**c**) Pearson’s correlation analysis was performed to analyze the correlation between the parameter estimates of the left SI and subjective pain ratings. We found significant correlation between the reduced left SI activities and decreased pain ratings (*r* = 0.50, *p* = 0.024). SI, primary somatosensory cortex; TPJ, temporoparietal junction.

To identify the neural mechanisms underlying the operant choice behavior, we investigated brain activity related to the cue-selection task in active learning trials compared to the activity related to the passive selection task during classical learning trials when participants clicked the cue previously determined by the computer. During the test session, the active cue-choice task evoked significantly greater activation in the left inferior parietal gyrus and superior temporal sulcus, the regions comprising the temporoparietal junction (TPJ), than did the passive selection task (peak coordinate: X = −45.5, Y = −60, Z = 56.5, 38 voxels, Z score = 2.89, cluster-level FWE corrected *p* < 0.05; Fig. 3a).

### Brain activity during pain perception (test session)

We modeled the brain responses to the second pain in the test session regardless of the type of learning using fMRI) and found that the left primary and secondary somatosensory cortices, primary motor cortex, right superior parietal gyrus, bilateral superior frontal gyrus, anterior cingulate cortex, thalamus, caudate, and anterior-middle insula were significantly activated, while activity in the bilateral lingual and fusiform gyrus was significantly reduced in response to the painful mechanical stimulus (Table 1).

**Table 1:** Brain regions showing increased or decreased activation in response to painful mechanical stimulation.

Activation	Clusters	Location (L/R/Bilateral)	Alpha	Size of clusters	Z score	Coordinates of peak voxel in MNI space		
						x	y	z
Increased activation	Cluster 1	SI (L)	< 0.01	2301	3.49	− 38.5	− 32	70.5
		MI (L)				− 42	− 11	67
		Superior frontal gyrus (Bi)				− 17.5	69.5	0.5
						24.5	− 4	70.5
		Anterior cingulate cortex (Bi)				0	20.5	− 10
						7	34.5	28.5
		Thalamus (Bi)				− 10.5	− 18	11
						14	− 14.5	11
		Caudate (Bi)				− 7	6.5	− 10
						10.5	3	11
		SII (R)				63	10	7.5
		Anterior-middle insula (R)				45.5	20.5	− 3
	Cluster 2	Superior/inferior parietal gyrus (R)	< 0.01	673	3.47	35	− 60	63.5
						56	− 32	56.5
		Superior occipital cortex (R)				31.5	− 81	46
	Cluster 3	SII (L)	< 0.01	283	2.81	− 56	10	0.5
		Anterior insula (L)				− 31.5	10	− 17
		Caudate (L)				− 7	6.5	− 10
		Thalamus (L)				− 10.5	− 18	11
Decreased activation	Cluster 4	Lingual gyrus (Bi)	< 0.01	1618	− 2.84	− 31.5	10	− 17
						14	− 49.5	− 6.5
		Fusiform gyrus (Bi)				− 28	− 11	− 38
						42	− 25	− 31

Clusters of brain regions where activity was significantly increased or decreased during mechanical pain stimulation in all trials regardless of learning type and visual cue during the test session. The results were corrected for multiple comparison at a cluster-level threshold of *p* < 0.05, and the cluster size was determined by permutations (n = 1000).

Bi, bilateral; L, left; MI, primary motor cortex; MNI, Montreal Neurological Institute; R, right; SI, primary somatosensory cortex; SII, secondary somatosensory cortex.

We compared brain activity in response to the second pain experience between the active and passive learning trials (operant learning trials vs. classical conditioning trials). Brain activity in response to the painful stimulus was significantly reduced in the left primary somatosensory cortex (SI, peak coordinate: X = −52.5, Y = −18, Z = 60, 94 voxels, Z score = −3.25, expanding to the precentral gyrus and parietal lobe) and the right lingual gyrus (peak coordinate: X = 17.5, Y = −53, Z = −13.5, 52 voxels, Z score = −4.15, cluster-level FWE corrected *p* < 0.05) during the operant learning trials relative to the classical conditioning trials (Fig. 3b).

Pearson’s correlation analysis was performed to analyze the correlation between parameter estimates in the SI [operant—classical learning trials] and subjective pain ratings [operant—classical learning trials]. We found significant correlation (*r* = 0.50, *p* = 0.024) between the reduced left SI activities and decreased pain ratings (Fig. 3c).

## Discussion

We investigated the pain modulation effect and the neural mechanisms involved in operant and classical conditioning as an explicit example of mind–body interaction. The behavioral results showed that (1) participants voluntarily selected visual cues paired with greater pain relief (UL and CL) significantly more than the visual cue paired with less pain relief (CH); (2) the predictive visual cue associated with greater pain relief (UL and CL) significantly reduced the reported pain intensity compared with the cue signaling less pain relief (CH) in both operant and classical conditioning trials; and (3) the pain ratings were significantly reduced in the operant learning context compared with the passive classical learning context. However, the amount of placebo-like effect (reduced subjective pain ratings in low-cue trials compared to those in high-cue trials) was not significantly different between operant and passive learning conditions. These results suggest that participants learned the predictive value of the cues through operant as well as classical conditioning, and placebo-like effects were induced by both types of conditioning. Moreover, the finding that participants rated significantly less pain in response to the same pinprick magnitude in instrumental conditioning trials than in classical conditioning trials suggests that pain perception was decreased more during the instrumental learning and active decision-making procedure. Consistent with the behavioral data, fMRI data revealed that activity in the SI in response to more intense pain was significantly reduced in operant conditioning trials compared with classical conditioning trials.

In the current study, the pain modulation effect in operant conditioning condition did not differ from that in classical conditioning condition, but overall pain reduction was greater in operant conditioning than classical conditioning condition. An increase or decrease in pain as a punisher or reinforcer, respectively, also modulated responses to pain. Operant conditioning allowing active choice of options followed by modified pain intensity^24,25,27,35,36^ or reports of reinforcing or punishing subjective pain induces the placebo effect^26^. Contingency between pain responses and rewards/punishers successfully induced placebo analgesia through operant conditioning paradigm^26^. Our previous study, which employed an operant conditioning paradigm, allowed an active choice of treatment options (doctor or pharmacy) that were associated with modulated pain intensity, resulting in greater pain reduction. We found that two reinforcers, which had different degrees and certainties of pain relief and cost, led to a significant modulation of pain compared to the absence of treatment control, but the changes in pain perception by the two reinforcers did not differ^35^. Janssens et al*.* investigated operant learning of the association between avoidance behaviors that modulated pain intensity but required differing degrees of effort. They found that operant learning changed expectations for pain and movement-choice behavior; however, the pain modulation effect was observed only for low-cost choices^36^. The effect of operant conditioning on pain perception in the two previous studies should be distinguished from the placebo effect by operant conditioning^35–37^.

Skinner suggested that most human behaviors are not elicited involuntarily as ‘conditioned responses’; instead, he argued that humans operate on their environment, and their behavior is instrumental in achieving certain consequences (e.g., gaining rewards)^38^. In this study, placebo-induced pain modulation was defined as a phenomenon in which low-pain predictive cue reduces the subjective pain experience compared with high-pain predictive cue. Although operant learning was not more powerful than classical learning in terms of the placebo-like effect in this study, it might be a more accurate model of the learning that underlies the placebo effect in clinics. Patients come to the clinic with a history of previous experiences with, and expectations of, a treatment, and their behavior (treatment choice) may change as a function of its consequences (clinical outcomes). In this paradigm, previous experiences of clinical outcomes (past consequences) convey important information to patients, reinforcing or suppressing voluntary behaviors (treatment choice). However, we are not arguing that patients’ clinical decision-making processes are solely based on operant learning. For example, patients are not usually exposed to various treatment options, and in a worse case, patients are excluded from medical decision-making processes and passively receive a treatment (ideally still they should be provided detailed information). Thus, interactions and interference between operant and passive learning processes are crucial to understand placebo effects found in clinical practice. Lastly, using pain relief as reinforcement also contributes to making our approach more similar to clinical settings, as we are more likely to experience it than we are to receive verbal reinforcement in clinics.

Operant conditioning is also called instrumental conditioning, and the instrumental action is based on two distinct systems: a goal-directed system that is sensitive to action-outcome contingencies and a habit system that is not sensitive to the incentive value of the outcome^39^. Especially for goal-directed learning, cognitive functions such as exploration, evaluation, and comparison of the value of given options, as well as motivation, are fundamental. In terms of pain, motivation distracts our attention from and alleviates pain^40,41^. In addition, in the context where the instrumental behavior is available, perceived controllability is enhanced, changing pain processing in the brain^42–44^. Taken together, processes such as motivation, controllability, and value comparison might be stronger during instrumental learning than during passive learning (i.e., classical conditioning); these processes could contribute to the reduction in pain ratings and SI neural activity in response to painful mechanical stimulation in active compared with passive learning trials.

In the present study, neural activity in the TPJ was significantly greater during the active choice of visual cues than during the passive clicking on cues selected by the computer. The TPJ has been studied mainly from the perspective of social neuroscience, and it is widely accepted that the function is right lateralized and is involved in empathy and perspective taking^45,46^, stimulus driven re-orientation of attention to a stimulus related to our behavior^47,48^, and goal-directed social behavior^49,50^. On the other hand, Geng and Vossel criticized these ideas and argued for the “contextual updating hypothesis,” which states that the TPJ is engaged in processing unexpected task stimuli, modulating expectations for outcomes of a behavior, and updating the context of human behavior, which is necessary for appropriate decisions. Also, Hackel et al*.* found that the right TPJ was significantly correlated with the prediction error of generosity in others (the proportion of shared money), but not with prediction error of absolute reward value (the magnitude of shared money) during instrumental learning^51^, implying that the TPJ is involved in processing prediction error (differences between what is expected and what actually happens). Although more evidence is required, we suggest that the role of the TPJ during active cue-selection in operant trials in our experimental paradigm is related to the explicit demands on attention for updating the context and the value of task stimuli in terms of their reward and (un)certainty. Future studies are required to address how dynamic changes in expectations about consequences and context influence the learning process and about the pain-modulating effects of operant conditioning.

At the early stages, many studies have shown that placebo effects induced by classical conditioning are mediated by expectancy^15^. However, expectancy may not always be involved in placebo effects induced by classical conditioning, and conditioning may be a distinct mechanism of placebo effect^16^. For instance, hidden conditioning without verbal suggestions was effective in producing placebo effect^52,53^. Moreover, placebo effects were successfully induced by conditioned stimuli provided subliminally without the awareness of the participants^54,55^. In the current study, participants were informed that they would receive the different types of electrical stimulation. Therefore, expectancy by verbal suggestion might be involved in the pain modulation effect induced by both classical and operant conditioning in this study. Although the verbal suggestions were given to all types of trials (CH, CL, and UL), thus the pain modulation effects by comparing the pain ratings of CH trials with those of CL and UL trials might not be directly derived from the conscious expectancy. Since the main purpose of the present study was to explore neural mechanisms underlying pain modulation effects of operant and classical conditioning paradigm, further studies are necessary to distinguish the pain modulation effect induced by conditioning paradigm from that by verbal suggestions in the future.

This study has some limitations. Our study demonstrated that participants selected low-pain cues more frequently than high-pain cues in operant learning trials. The number of cue choices might be one of the indices of the learning process of operant learning in the current study. However, the different numbers of trials between operant and classical conditioning might confound our results. As we used only the high pain in the test session, the stimulus intensity that participants received during operant conditioning trials was the same as the intensity that they received during classical conditioning trials regardless of the number of trials. Nevertheless, our results might have been biased since we did not match the same number of trials between operant and classical conditioning. Future study should carefully consider the number of conditioning trials when comparing the two different conditioning types. Second, our study used the magnitude estimation method to measure the degree of pain, which inevitably requires a given reference pain prior to the cue. On the other hand, as participants were presented the reference pain in each trial, we were able to minimize the effect of time on their subjective pain ratings (e.g. sensitized or habituated pain response). Given that operant conditioning establishes functional relations between behaviors, its consequences and antecedent stimuli^37^, it can be appropriate to apply the magnitude estimation method to measure the changes of pain level after making decision. As we did not measure behavioral responses directly, it was not able to analyze the relationships among behavioral responses (e.g. expectancy of pain reduction and reduced pain reports) and between behavior and brain activities, other than the correlation of subjective pain ratings with brain activities in the left SI. Lastly, the sample size was quite small in the current study. Researchers raised the concerns in which relatively low power of fMRI studies contribute to an increased risk of false or exaggerated results^56^. More sufficient data are needed to overcome the reproducibility of this study.

In conclusion, we have shown that both operant and classical conditioning successfully induce placebo-like effect. Operant behavior, defined here as the ability to choose visual cues predicting upcoming painful events, resulted in increased activation in the TPJ, suggesting that instrumental learning requires the mechanisms of attention, context change, and prediction error of expectation of behavioral consequences. The reduced activity in the SI during the pain period in operant learning trials demonstrated that manipulation of the learning process altered the neural response to pain as well as reducing behavioral pain intensity ratings. The present operant learning paradigm provides a useful tool for further investigation of the effects of learning the reward value of the pain-relief cue on instrumental behavior and on pain modulation. Investigating pain modulation using an operant conditioning model will contribute to our understanding of the patients’ minds and behaviors in clinical practice and deepen our understanding of the mind–body interaction.

## Methods

### Participants

A total of 21 healthy volunteers (right-handed, age: 24.1 ± 4.5 years; 12 females) were recruited. None of the participants had any history of neurological, psychiatric, or other major medical problems, and none was taking medications at the time of the study. All subjects filled out informed consent forms to take part in the study. This study was approved by the ethics committee of Korea University (IRB no. 1040548-KU-IRB-15-250-A-1) and conducted in accordance with the guidelines of the Human Subjects Committee of Korea University. We calculated the size of sample based on our previous placebo study, in which we measured placebo response during instrumental conditioning between control trials and learning trials^35^. The effect size *d* of pain modulation effect between control and learning trials was 1.076, and thus we need total 42 participants for two-sided test, alpha level 0.05. As a pilot study to test feasibility and examine preliminary evidence, we have 21 healthy participants in this study.

### Experimental design and procedure

#### Instructions and experimental paradigm

Prior to the experiment, participants were given the following instructions: “This study will assess a newly developed analgesic device called the Transdermal Micro-electric Current Stimulation Device (TMCSD). This device is a novel technique that will provide analgesic effects, which varies according to the TMCSD electrical stimulation type.” The TMCSD was a sham device that did not deliver any electrical stimulation. Participants received all information through one experimenter’s reading written instructions; they received no other information, including regarding the placebo effect.

After consent was obtained, participants completed a 2-day experiment. At the beginning of each visit, the TMCSD device was attached to the dorsum of the right foot. Participants were asked to lie on a bed and to press buttons for pain ratings. On day 1, participants were subjected to a conditioning session to familiarize them with the task. They were instructed to evaluate the analgesic effects of imperceptible electrical stimulation produced by the TMCSD. Visual cues paired with the electrical stimuli appeared on a screen while participants thought they were receiving the corresponding electrical stimulation. On day 2, fMRI was carried out while the participants were undergoing a conditioning session (as on day 1) and an additional test session.

To evaluate the analgesic effects, painful mechanical stimulation was applied to the back of the left hand twice: (1) the first painful stimulation, applied using a 512 mN pinprick needle (weighted needle-shaped, non-penetrating mechanical stimulation; MRC Systems, Heidelberg, Germany), served as a reference (reference pain) at the beginning of the trial; (2) the second stimulus, administered by a 32, 64, 128, or 256 mN pinprick needle, evoked the second experience of pain based on the selected visual cue, which represented the amount of analgesic effect they would receive. All pinprick stimulations were delivered by an experimenter according to the beat of a 1-Hz metronome transmitted via headphone. To compare the pain modulation effects and neural mechanisms of the two conditioning procedures, the participants underwent both a classical conditioning and an operant conditioning session. We expected that the participant’s expectation for the cues predicting the analgesic effect of the device would be shaped by the repeated learning procedure, where the intensity of the second pain stimulus was always lower than the first stimulus, but with varying degrees of pain relief. During the operant conditioning session, participants could select different types of electrical stimulations. In the classical conditioning session, participants received pre-defined sets of electrical stimulations and were not allowed to choose the cues. At the end of each trial, participants evaluated the relative pain intensity of the second stimulus against the reference pain. As the verbal suggestion about the TMCSD might produce analgesic effect, we defined the placebo-like effect as ‘reduced subjective pain ratings in low-cue trials and those in high-cue trials’. However, it might not be appropriate control to measure pure placebo effect. Since the verbal suggestion affects all types of trials (CH, CL, and UL), we could identify the effect of cue on their pain ratings by comparing the pain ratings of CH trials with those of CL and UL trials.

#### Conditioning and test sessions

**Figure 4:**
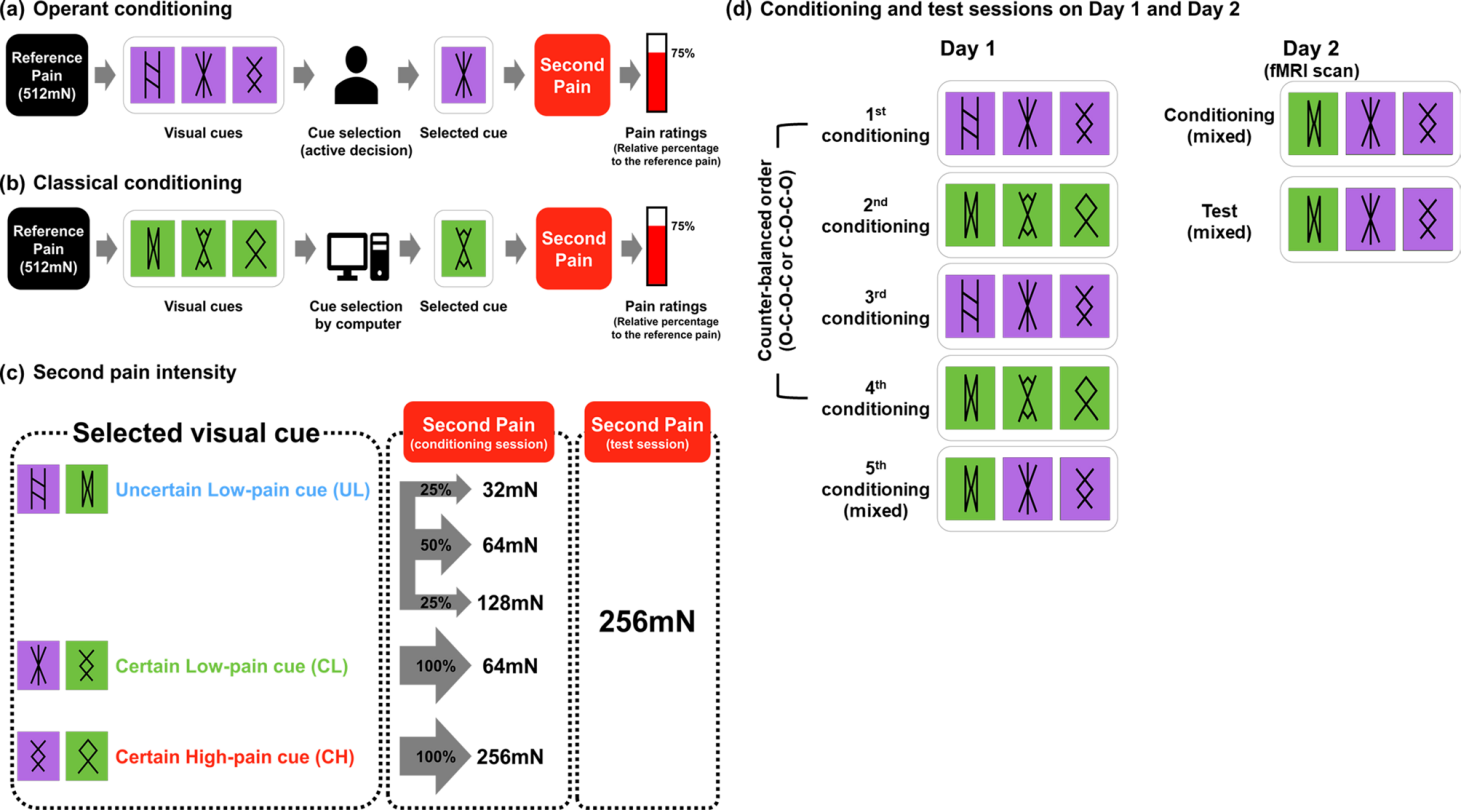
Illustration of the experimental process of operant conditioning and Pavlovian (classical) conditioning trials. (**a**) Operant conditioning trial: a mechanical stimulus with reference intensity of 512 mN was applied for 2 s, and one of three visual cues comprising *Lun* characters on the same background color (purple and green, counterbalanced) was shown on the screen for 4 s for selection by participants. Participants were told that they would receive three different electrical stimuli known to have different analgesic effects indicated by the selected cue. After the participant chose a character, the selected cue was displayed on the screen for 2 s, and the second painful stimulus was delivered for 2 s. At the end of each trial, participants evaluated the intensity of the second experience of pain relative to the reference pain for 6 s. (**b**) Classical conditioning trial: all procedures were the same as for the operant conditioning trial except that the cue was determined by a computer before the beginning of the session. Participants were asked to click on the computer-selected cue for 2 s when it appeared on the screen. (**c**) Stimulus intensity for the second pain experience according to the selected visual cue and session (conditioning type and test). For the purpose of forming an association between the visual cues and pain intensity during the conditioning session, three types of mechanical pain were delivered using a weighted needle. If participants chose the uncertain low-pain (UL) cue, a 32, 64, or 128 mN pinprick needle was applied at probabilities of 25%, 50%, and 25%, respectively. If participants chose the low-pain (CL) or high-pain (CH) cue, a 64 mN or 256 mN pinprick was applied, respectively. A reference pain (512 mN) was used to measure the relative magnitude of the second pain using a magnitude estimation method. During the test session, the second painful stimulus was always delivered using a 256 mN pinprick needle to measure the placebo analgesic effect induced by modulated expectations for upcoming pain levels conditioned to the three visual cues. (**d**) On day 1, the participants underwent two classical conditioning, two operant conditioning, and one mixed conditioning session. In the first four conditioning sessions, participants experienced only one type of learning in each session according to the counter-balanced order (operant–classical–operant–classical or classical–operant–classical–operant). In the last conditioning session of day 1, conditioning and test sessions of day 2, participants experienced both types of learning (mixed) to eliminate order effect during the test session. O, operant conditioning; C, classical conditioning.

On day 1, the participants underwent two classical conditioning, two operant conditioning, and one mixed conditioning session (Fig. 4d). Participants were informed that they would first receive a mechanical stimulus with a non-penetrating needle (the reference pain using a pinprick weight of 512 mN), and then look at one of three ancient *Lun* characters with two different background colors (purple or green). The background colors indicated which task they were performing, either classical or operant conditioning. The three *Lun* characters indicated three electrical stimulation types, which were not actually delivered. The three *Lun* characters served as predictive cues for upcoming pain relief: the uncertain low pain (UL) cue, the certain low pain (CL) cue, and the certain high pain (CH) cue. The UL cue was followed by the 32, 64, or 128 mN weighted pinprick needle at probability rates of 25%, 50%, and 25%, respectively. The CL and CH cues were followed by a mild (64 mN) and high (256 mN) level of pain, respectively (Fig. 4c). Assignment of the *Lun* characters and background colors was randomized and counterbalanced across participants. Participants could not see the administration of the pain stimulus, as it was hidden by a board. Subjective pain ratings were obtained using the magnitude estimation method, in which participants rated the relative (percent) magnitude of pain induced by the second pain stimulus relative to the first, reference stimulus^35^.

The operant learning task allowed participants to freely explore, experience, and evaluate the pain modulation effects of three visual cues to generate cue-based expectancies. On the other hand, the classical conditioning task permitted participants to learn the association between the cues and stimulus intensities passively by pre-defined sets of trials. After viewing the cues, participants were asked to press a button to choose a cue (operant learning) or to click a pre-selected cue (classical conditioning; Fig. 4a,b). The visual presentation was programmed using the Psychtoolbox program in Matlab (MathWorks, Natick, MA, USA). There were nine trials for the classical conditioning and three learning trials for each visual cue; the trial number for the operant conditioning session was dependent on the participants’ choices, except that they would experience all visual cues at least once and the total number of trials was restricted to 6–12. The mixed session included 12 classical learning trials and 8–16 active learning trials.

On day 2, a test session following a mixed conditioning session was conducted in the MRI scanner (Fig. 4d). The test session was similar to the conditioning sessions, except that only the high-intensity pain (256 mN) was delivered as the second stimulus to test the effect of cue learning on pain perception.

On day 1, there were 5 conditioning sessions, two for operant learning and two for classical conditioning, for the purpose of practicing the tasks. The order of the first 4 conditioning sessions were either operant-classical-operant-classical or classical-operant-classical-operant, counter-balanced between the participants. The last conditioning session was always a ‘mixed conditioning session’, in which the two types of trials were randomly ordered. On day 2, operant and classical conditioning trials were randomly intermixed (mixed conditioning session only). The study protocol was designed to help the participants learn two different tasks separately and to minimize the order effect during fMRI scans.

Throughout the first 4 conditioning sessions, where the types of trials were clearly distinct between sessions, participants could practice the study paradigm and separate two types of learning process easily. The last mixed conditioning session of day 1 served as a practice session for the conditioning session of day 2, which was also a mixed conditioning session. On day 2, inside of the scanner, participants performed the two types of learning in randomized order, so that we could exclude the order effect and prevent the participants from being habituated.

### Brain imaging acquisition

The methods described below have been reproduced in part from Lee et al*.*^57^. fMRI scans were acquired using a Trio 3 T scanner (Siemens, Erlangen, Germany). Blood oxygen level-dependent fMRI of the whole brain was assessed using an echo planar imaging (EPI) sequence (TR = 2000 ms, TE = 30 ms, flip angle = 90°, field of view = 240 × 240 mm^2^, voxel size = 3.8 × 3.8 × 4.0 mm^3^, 37 slices). As an anatomical reference, a T1-weighted image was obtained using a magnetization-prepared rapid gradient echo sequence (TR = 2000 ms, TE = 2.37 ms, flip angle = 9°, field of view = 240 × 240 mm^2^, voxel size = 0.9 × 0.9 × 1.0 mm^3^, 192 slices).

### Brain imaging analysis

Preprocessing was performed with Analysis of Functional NeuroImages software (AFNI version 19.2.24, https://afni.nimh.nih.gov). EPI data were corrected for slice timing and motion, concatenated, and transformed to a Montreal Neurological Institute (MNI) template space, registered to the volume with the minimum outlier fraction, spatially blurred using a 4-mm full-width at half-maximum (FWHM) Gaussian filter, and scaled to a mean of 100 for each voxel.

fMRI data were analyzed at the individual subject level using the input from all stimuli in a multiple linear regression using a gamma variate hemodynamic response function convolved with a boxcar function. Contrast images corresponding to cue selection and painful stimulation in operant and classical conditioning sessions were generated. The cluster threshold was determined by Monte Carlo simulations, and results were evaluated using a family-wise error (FWE) corrected significance threshold of *p* < 0.05^58^. The actual smoothness of the data was determined using 3dFWHMx, with an auto-correlation function for each participant. Then, the mean FWHM value across participants was used in the 3dClustSim program with an uncorrected threshold of *p* < 0.005 for volume correction simulations.

### Statistical analysis

All statistical analyses were conducted in R using a linear mixed-effect model including subject as a random effect to account for inter-subject variations. We evaluated the learning of cue-based expectancies using the ratio of trials in which each visual cue was selected (UL, CL, and CH) in operant conditioning trials using a repeated-measure analysis of variance (ANOVA) and post hoc with Bonferroni test. A two-way ANOVA and post hoc *t*-tests with Bonferroni correction were conducted for the pain intensity ratings of the second pain experience in the conditioning and test session on day 2, with conditioning type (operant and classical) and visual cue (UL, CL, and CH) as within-subject factors.
